# A robust, melting class bulk superhydrophobic material with heat-healing and self-cleaning properties

**DOI:** 10.1038/srep18510

**Published:** 2015-12-18

**Authors:** S. Ramakrishna, K. S. Santhosh Kumar, Dona Mathew, C. P. Reghunadhan Nair

**Affiliations:** 1 Polymers and Special Chemicals Group, Vikram Sarabhai Space Centre, Thiruvananthapuram-22.

## Abstract

Superhydrophobic (SH) materials are essential for a myriad of applications such as anti-icing and self-cleaning due to their extreme water repellency. A single, robust material simultaneously possessing melt-coatability, bulk water repellency, self-cleanability, self-healability, self-refreshability, and adhesiveness has been remaining an elusive goal. We demonstrate a unique class of melt-processable, bulk SH coating by grafting long alkyl chains on silica nanoparticle surface by a facile one-step method. The well-defined nanomaterial shows SH property in the bulk and is found to heal macro-cracks on gentle heating. It retains wettability characteristics even after abrading with a sand paper. The surface regenerates SH features (due to reversible self-assembly of nano structures) quickly at ambient temperature even after cyclic water impalement, boiling water treatment and multiple finger rubbing tests. It exhibits self-cleaning properties on both fresh and cut surfaces. This kind of coating, hitherto undisclosed, is expected to be a breakthrough in the field of melt-processable SH coatings.

Superhydrophobic (SH) surfaces have been captivating the researchers and industrialists evenly owing to their excellent water repulsion and self-cleaning properties^1^,^2^,^3^,^4^. To the attribute of outstanding water blocking capability, they find exotic applications such as anti-bacterial, anti-corrosion, anti-icing, self-cleaning and similar kind of several functions^4^,^5^,^6^,^7^,^8^,^9^. Lotus leaf which exemplifies the superhydrophobic behaviour from nature comprises micro and nano roughness on its surface structure (along with hydrophobic wax). This rare combination is the rationale for high water contact angle and water rolling properties of lotus leaf. Subsequently, enormous research efforts were dedicated to prepare SH surfaces by utilising the basic combination of micro-nano roughness and hydrophobic chemistry^10^,^11^,^12^.

SH surfaces can be prepared by electrospinning^13^, microlithography^14^, photolithography^15^,^16^ and etching techniques^17^. But these methods require highly sophisticated instruments and more control. Chemical vapor deposition is another route but it generally needs elevated temperature (>250 °C)^18^. Casting^19^, molding^20^, template^21^ and phase separation^22^ are other approaches for preparation of SH surfaces. However, either sophisticated methods or multi-step procedure or both are generally required in most cases^23^,^24^,^25^. For e.g., a multi-step method was reported, which involves the synthesis of polyurethane foam, coating it with dopamine-modified carbon nanotubes, self-polymerisation of dopamine, and finally conjugation of octadecyl amine onto poly (dopamine) sponge surface to achieve a water contact angle of 158°^23^. By atom transfer radical polymerization of 2-(N-ethyl perfluorooctanesulphamido) acrylate, SH polymer with good abrasion resistance was prepared by a three step method^24^. In another work, a superhydrophobic thin film was attained by deposition of a precursor, followed by charging hollow silica nanoparticles/ mesoporous silica nano sheets and finally subjecting to calcination/surface treatments^25^. Deng *et al.* prepared a macroporous SH membrane by hierarchical alumina template wetting method, where poly (methylmethacrylate) was treated with 1H,1H,2H,2H-perfluorodecyltriethoxysilane^26^. Considering facile step preparations, a limited number of reports are available. A large area SH coating was developed by blending TiO_2_ nanoparticles with α, β bis (hydroxylpropyl)-terminated fluorinated polysiloxane oligomer and α, β-bis (hydrogen)-terminated poly (dimethylsiloxane)^27^. Zhu *et al.* realized strongly adhering SH coating on polyurethane sponge, in a single step immersion process using methyl trichloro silane^28^. An interesting single step SH coating was achieved by electrospraying of polycaprolactone, however, low adhesion to the substrate was noticed^29^. Recently, self-cleaning SH surface was prepared from titanium dioxide nanoparticles, encapsulated using perfluorosilane moieties^9^. However, a facile and single step process is still in demand for all quarters of application^29^.

Superhydrophobicity at the surface level was the only cynosure in the beginning of SH surface research. The concept of bulk SH materials (SH property throughout the bulk of the material) has emerged recently^30^,^31^,^32^,^33^,^34^,^35^. Grinstaff *et al.*^30^,^31^ prepared three-dimensional SH coatings *via* different approaches like electrospinning and electrospraying. A mixture of poly (ε -caprolactone) and poly (glycerol monostearate-co-ε- caprolactone) was electrospayed to result a bulk SH coating on different substrates^30^. In another report, bulk superhydrophobic materials were accomplished by electrospinning poly (ε -caprolactone) and poly (glycerol monostearate- co - ε - caprolactone) mixture along with stearic acid^31^. A damage tolerant superhydrophobic and superoleophilic bulk SH material was realised by combining titania nanorods, silica nanoparticles and polydimethyl siloxane^32^. We achieved bulk level SH properties in calcium carbonate based coating using silane and urethane crosslinking chemistry, which resulted in excellent bulk SH feature with water rolling surface (water roll off <8°)^33^. A combination of polytetrafluoroethylene, carbon nanotubes and iron nanoparticles were also reported as a bulk SH material^34^. Similarly, by blending polytetrafluoroethylene and carbon nanotube at 390 °C, a bulk SH material was obtained. In this case, the SH property was recoverable, but heating at elevated temperature is needed (at 500 °C)^35^. For oil –water separation, several porous SH materials with bulk level water repellency were reported elsewhere^23^,^28^,^36^,^37^,^38^,^39^.

Most of the SH coatings are fragile to mechanical stress and are not durable either. This concern was investigated by different groups^40^,^41^,^42^. Hua Zhou *et al.*^40^ reported a mechanically durable superhydrophobic coating on a fabric material where the coating was prepared from a blend of modified silica nanoparticles and polydimethylsiloxane. The SH property of the coating could be recovered on drying at ambient conditions (24 h) after acidic and basic solution treatments. In another approach, a disc shaped, mechanically durable SH material was obtained by pressing ultra-high molecular weight polyethylene and copper powder, followed by deposition of Ag film and perfluoro decanethiol^41^. Recently, mechanically robust SH coating was realized on cotton fabric by depositing TiO_2_ microparticles and fluoroalkylsilane^42^.

As the SH materials are soft coatings, it is very difficult to maintain the SH properties after severe acid/base/mechanical attacks due to the damage of the micro-nano roughness. But, if they can refresh the surface, they will become very promising. Hence, self-refreshable surfaces are unique as they regenerate SH properties even after harsh and multiple environmental attacks^43^,^44^. Such phenomena are observed in nature too. For e.g., after subjecting living clover leaves to plasma environment, SH properties were lost, but the SH features were recovered at ambient conditions within 48 h^43^. However, the self-recoverability was not observed with non-living clover leaves. Recently, we prepared self-refreshing SH particles and coatings by building urethane oligomers on silica surface^44^. Bringing self-healability/self-recoverability (i.e. regeneration of SH property of surfaces) add value to any material and these kind of SH coatings have been achieved primarily *via* fluorine and silane chemistries^45^,^46^,^47^,^48^,^49^,^50^,^51^,^52^. A combination of perfluorinated-decyl and fluorinated alkyl silane brought self-healing property to SH cotton fabric where healing could be achieved by heating the fabric at 135 °C for 5 minutes or by maintaining at ambient conditions for 24 h which permit the fluorinated chains to reach the surface to facilitate self-healing^45^. Recently, SH property was regenerated by UV light irradiation where fluoroalkylsilane loaded micro capsule healing agents were migrated onto the surface on irradiation^46^. Sun *et al.*^47^,^48^ reported fabrication of a self-healing SH cotton fabric by a two layer coating approach. The lower layer consisted of ammonium polyphosphate/ poly (ethylenimine) mixture and top layer was made of perfluorodecane thiol functionalised POSS. Though the surface of fabric becomes hydrophilic on O_2_ plasma treatment, the coating transformed into superhydrophobic due to the enrichment/floating of perfluorodecanyl chains over the surface. Here, the self-healing capability depends on the content of self-healing agent which is evident from the result that, on 10^th^ cycle of O_2_ plasma treatment, recovery time increased to 4 h *vis-a-vis* 1 h of 1^st^ cycle. In another report, layer-by-layer assembly of polyelectrolyte complexes of poly (allylamine hydrochloride), sulfonated poly (ether ether ketone) and poly (acrylic acid) resulted in SH property with self-healing features after chemical vapour deposition of fluoroalkylsilane healing agent^48^. In all these cases, self-healability was achieved by employing healing agents (reservoir for healing) and hence the extent of self-healability or recoverability on repeat cycles is limited. If an SH coating is healable by melting alone, such an SH material can exhibit unlimited number of self-healing cycles without any loss of SH properties.

While making artificial SH surfaces, some of the vital features of natural SH surfaces may get lost. For example, self-healing/self-refreshing property of lotus leaves is possible in living condition only, whereas the artificial SH surface relies on the content and nature of self-healing agent as reported hitherto (in most of the previous works). It is a challenge to make artificial SH materials identical to a natural surface. This work discloses our attempts in this direction. Despite previous efforts, a single material/coating possessing melt-processability, self-healability, self-cleanability, robustness and bulk level superhydrophobicity simultaneously has not been achieved. We attempt to realize such a unique material *via* grafting of long alkyl chains on silica nanoparticle surface which enables the resulting alkyl chain-grafted nano silica powder to undergo melting and to attain bulk level superhydrophobic coating. A mechanism responsible for regeneration of SH properties is also described. The bulk SH property, self-cleanability, heat-healability and surface robustness of the material are demonstrated.

In this work, octadecyl chain grafted silica nanoparticles (ODS18) were synthesized *via* silanol-isocyanate reaction in a one-step method (at 80 °C/18h) and characterized thoroughly (Figs 1a and *S1* for materials, methods and characterization). Differential scanning calorimetry exhibited an endotherm at 120 °C which implies the melting nature of grafted nanoparticle. Previously, melting of octadecyl chains (on polymerised n-octadecyl siloxane nanosheets) was observed at 80–90 °C^53^,^54^,^55^, at 79 °C for octadecyl phenyl urethane and at 116 °C for methylene bisoctadecyl urethane end molecules^56^,^57^. The increased melting point here is due to the enhanced crystallisation of the octadecyl groups, directed by the closely packed urethane groups. The melting and formation of crystals of octadecyl chains were clearly visible in the optical images by a progressive heating to 150 °C and subsequent cooling (Fig. 1d). The higher melting point implied possibility for the alignment of the long alkyl chains to form crystalline structures on silica surface (as in lotus surface). The crystalline nature of octadecyl moieties on SNP was further confirmed by XRD *(S2).* Most strikingly, the absorption of carbonyl group appeared at a very low frequency of 1614 cm^−1^ in FTIR which is attributed to the highly dense, strongly hydrogen bonded urethane species on the silica surface.

We prepared a coating of ODS18 particles by heating its colloidal solution in xylene at 150 °C for 8h on a glass slide by drop-casting method. The light yellowish particles fused themselves to a uniform and opaque coating on glass surface. The coating displayed a static water contact angle (WCA) of 166 ± 1° with a sliding angle ~6° (*S3*, for video of rolling/bouncing water droplets on SH surface). The surface energy was estimated as 10.5 mN/m (Harmonic mean method using three liquid approach, static contact angle of ethylene glycol was 136.5 ± 1°and diiodomethane was 62 ± 2°). Also, the ODS18 surface showed no substantial change in water repellence over a wide range of pH (1-13), where the static WCA values were >155° (Fig. 2). To the best of our knowledge, silica particles capable of melting and forming superhydrophobic coating have so far not been reported. Such a unique material can find industrial applications i.e. wider area application by powder coating. In literature, most of the SH surfaces need fluorinated molecules due to the excellent water repelling feature of fluorine^9^,^45^,^47^,^48^. In this backdrop, the present material is highly important that it does not contain any expensive fluorine molecules and is prepared by a one-step process.

Durability of surface is always a concern in SH coatings as micro-nano roughness can easily be destroyed by minor force/impact. Hence, many researchers focussed their studies primarily to address this point. To see the durability of our SH coating, three tests were carried out viz; i) water impalement for 20 cycles (one cycle: impaling SH surface with tap water for 120 s at a height of 50 cm with a water flow rate of 80 ml/s, and keeping at ambient condition/heating for self-recovery) ii) immersion in hot water (70 °C) for 20 cycles (one cycle: immersion in boiling water for 5 minutes and keeping at ambient condition/heating to 70–80 °C for self-recovery) and iii) rubbing test for 300 times.

Immediately after the water impalement, the ODS18 surface lost its superhydrophobicity, but, regenerated the SH features within 2 h at ambient conditions, after keeping it away from the source of water. The static WCA values were well above 150° till the last cycle (Fig. 3a), but the roll-off angle increased marginally. However, it was maintained below 10° (i.e. Cassie-Baxter state: If water droplets adhere to the surface even if static WCA is >150°, the SH state is called as Wenzel state (e.g. rose petal). If water droplets roll-off from the SH surface (instead of adhering), the state is called Cassie-Baxter state (static WCA >150° and roll-off angle <10° e.g. lotus leaf)). Nevertheless, the time needed to recover SH property increased with number of cycles and was 24 h (at ambient condition) at 20^th^ cycle. In a separate experiment, on heating at 80 °C, superhydrophobicity was found to be regenerated within 5 minutes during the initial cycles which extended to 15 minutes on 20^th^ cycle. Figure 3b shows the FESEM images where the crystalline features of the ODS18 coating before and after water impalement can be seen. The crystalline regions were very closely packed before the test of water impact. Most likely, the array of alkyl chains shifted from their equilibrium position due to water impact which facilitated the entry of water droplets to the proximity of the polar –O-CONH groups. Consequently, wetting increased after water impalement test. On heating, the flexible, long chains re-assemble themselves to regain SH property. Further, the optical microscope analysis showed glowing regions on the ODS18 coating on both the original and self-recovered surfaces, which implies that the crystalline nature was maintained after the tests *(S4).* It may be noted that lotus leaf also contains long alkyl chain and crystalline domains accounting for their SH property (similar to the present material).

Previously, hierarchical structures of hairy carbonaceous fiber exhibited SH property which was prepared by chemical vapor deposition of acetylene gas on different substrates at 230–300 °C^18^. Also, SH property was induced on hydroxyethymethacrylate (HEMA)/glycerol dimethacylate films by dense hairy structures (or ultra-long micro fibre, using mammalian teeth template)^58^. Similar to these works, needle or hairy like structures are the rationale for superhydrophobic properties in the present coating too. Several other nanomaterials also have been explored for realizing SH materials elsewhere. Recently, two beam laser interference treatment by laser holographic method on graphene oxide films created hierarchical roughness and imparted SH properties to graphene film^59^. Self-assembly of gold nanoparticles with fullerene pyridyl derivatives resulted in thin superhydrophobic films. These films display self-repairing property from physical breakage in the presence of toluene solvent^60^. But, single step functionalization below 100 °C, bulk SH properties and most important property of melt-coatability observed in the current coating were lacking in those systems.

The general rationale for superhydrophobicity is increased roughness (at nanometres level) on the coating where air can be trapped effectively. The average surface roughness of the present coating got reduced from 70 nm to 60 nm on water impalement as noted in AFM profiles (Fig. 3c). In this work, the applied velocity of water impalement was 3.13 m/s and the calculated water hammer pressure was 940 kPa *(S5)*. This impact pressure is higher than those reported in previous works, including SH silicon nanowires, textured SH surfaces and SH graphitic surfaces, which were tested with impact pressure of 35 kPa, 2 kPa and 75–315 kPa respectively^61^,^62^,^63^. In earlier reports, paraffin candle soot layer coated with silica shell and fluorosilane could not withstand even a low impact velocity of 1–1.5 m/s and all properties were lost after the water hammer test^64^. The water droplets adhered to the surface after the water hammer test in the case of graphitic SH surfaces^62^. In a recent report, SH film made of mesoporous silica nanosheets sustained water hammering of 5000 water droplets of size 22 μl (i.e. total volume of water falls is only 110 ml) for one test only^25^. In the present case, ODS18 withstood 9.6 litres of water impact with a velocity of 3.13 m/s in one cycle alone.

On this SH coating, the wetting phenomenon was not observed quickly after water impalement. However, wetting was observed immediately after immersion in boiling water (70 °C for 5 min). This is because, the trapped air between the needle like projections is expelled by the contact of liquids which is a common phenomenon observed in all SH systems. The contact of liquid creates pressure on the trapped air and the liquid can spread easily between the micro-nano roughnesses. As expected, after boiling water test, the SH property was recovered by gentle heating at 50–70 °C for about 15 minutes, On 20^th^ cycle, the surface took about 30 minutes for regenerating the SH property (water CA is 154°, roll-off angle <10°). The SH coatings that can sustain in boiling water are rarely reported^40^,^44^ .A super durable SH fabric retained superhydrophobicity after 5 h hot water test^40^. A PDMS-superhydrophobic silica coating also withstood 5 h hot water test^44^. But, cyclic, boiling water test durable SH coatings are rarely achieved.

Normally, the delicate SH property is vulnerable to finger contacts and finger-touches^65^. In the current work, we carried out a rubbing test by continuous manual finger rubbing for 300 times in the horizontal direction (pressure on each rubbing is around 10 kPa). The static WCA got reduced to ~162° on 100^th^ rubbing cycle, to ~158° on 200^th^ and to ~152° on 300^th^ cycle; starting from an initial static WCA of 166° of fresh surface. But the reduced WCA values are well above the superhydrophobic threshold limit (>150°). We noticed similar reduction in rolling capability of water droplets on the rubbed surface and the surface turned into Wenzel state with a roll-off angle of 20° on 300^th^ number rubbing test (6° for fresh surface). This is attributed to the partial loss of micro-nano roughness on the surface resulting in the Wenzel state. But, the SH properties could be easily regenerated by heating for several minutes at 70 °C even after 300^th^ rubbing test. We carried out a test to check the capability of the coating to regenerate the properties under contaminated condition. Firstly, the coating surface was subjected to multiple finger rubbing (the contact angle of the coating prior to rubbing was 162°), then dust particles were poured over the surface and heated the coating. Later, by pouring water over the surface, the dust particles got removed (self-cleaning). The contact angle of the coating was measured as >155° and water droplets were rolled-off with sliding angle <10° after this test. As the pollutant was present only on the surface of SH material and could not enter between micro/nano rough projections, the regeneration was not affected (See *S6*). Previously, Ag film deposited polyethylene surfaces and Ag film deposited polyester fabric (treated with perfluoro thiol) were reported to retain SH properties after a single finger touching test (but not with continuous rubbing)^41^,^66^.

As ODS18 is constituted practically of a single chemical structure, it can be expected to exhibit bulk level SH properties. The bulk material having dimension 4.0 cm length, 2.5 cm breadth and 0.7 cm height was prepared in a rectangular mould by heating the ODS18 powder at 150 °C for 8h. The topmost layer exhibited a WCA of 165 ± 1° with a sliding angle of ~6°. The bulk material was broken into pieces and tested for wettability. The WCA on the broken portions was noted as 160 ± 1° with a roll-off ~6°. Earlier reports speculate bulk superhydrophobicity that was achieved mainly by sophisticated techniques^30^,^31^,^32^,^33^,^34^,^35^. Since it is a meltable SH material, the broken portions could be joined together by heating (heat-healable). To prove this, the broken pieces were kept together in a mould and heated at 150 °C for 2h. The broken portions got joined perfectly (Fig. 4b). The static WCA was observed on the healed area was164 ± 1° with roll-off angle ~6°. Thus, the cracks could be healed effectively without disturbing SH properties. Further, the durability of healed bulk material was tested by abrasion with a sand paper (grit size P 100 i.e. particle size about 160 μm). The top surface (surface 1) of the bulk material was abraded with the sand paper with a pressure of 10.5 kPa (and this pressure is higher than other reported works^35^,^41^,^48^, abraded area = 7.5 cm^2^) and removed after 10 times of unidirectional abrasion to expose surface 2. The procedure was repeated 9 more times to remove the penultimate layers in each case and to get fresh surfaces numbered 3 to 11. Each surface after abrasion manifested the static WCA values between 159–162° with sliding angles of 8–10°, similar to the original coating (and the superhydrophobicity was retained till the last layer). This new SH material is inherently self-healing (to the best of our knowledge, first in the class of melting SH) and brings advantage that no fluorine chemistry or additional chemical reactions are required for achieving healing capability. As this material is a melt-healable, its self-healing nature is repeatable for several times (eternal self-healing).

Self-cleaning is a fascinating property for SH materials where high water repellence and low roll-off angle are essential. The fast rolling water droplets can clean the coating very effectively by catching hydrophilic dirt particles from the surface of materials. The self-cleanability of ODS18 coating was tested by pouring magnesium powder on the surface coated over glass slide. Water droplets were placed on top portion of slanting glass slide (angle <10°). As seen in the Fig. 4e (i–iv), hydrophilic powders got removed along with water droplets and a clean surface could be obtained. More interestingly, the self-cleaning nature of the surface was manifested even on scratched surface (horizontal and vertical cuts) made with knife (Fig. 4e (v)). Earlier methods employed fluorination route for this^9^,^67^.

In general, the SH coatings show poor adhesion with many substrates. In such cases, normally, commercial adhesives are used to develop good adhesion between the coating and the substrate^9^. Previously, fluorinated silica nanoparticles showed very good adhesion to tin plate and glass^68^. The adhesion of ODS18 coating with glass (Micro slide), stainless steel (AISI 304) and aluminium (AA2014) alloy was tested by cross-hatch method using a sharp knife. The area of SH coating chosen for testing was 1 cm^2^. On the pull-off test, the coating proved good with 95% retention on glass, 85% on SS while the bonding was not so good on AA 2014 substrate (retention of coating area was only 60%) (*S7)*. From the above tests, SH coatings of ODS18 proved themselves sustainable, durable and self-refreshable. It is also noteworthy that ODS18 surface exposed for one year under ambient conditions still retains its SH properties *(S8).*

We destroyed the surface roughness of ODS18 silica particles completely by applying a very high static pressure (of 5700 kg/cm^2^, pressed in a pelletizer for 120 s) to result a pellet (this way, contact angle due to chemical structure alone can be measured). Thus, with this pressure, the dual scale roughness (micro-nano) got reduced to 10 nm (AFM, *S9a*) from the original surface average roughness of 70 nm (Fig. 3c). In addition, the surface morphology was also altered due to the high pressure and no morphological features were identified from FESEM analysis *(S9b)*. The static WCA of the pressed surface was 106° which can be considered as the static WCA of the base material *(S9c)* and subsequently, the void volume was calculated (*S10)* and was found to be 96-91% for pH from 1 to 13*(S9d)*. Also, this pressure tested ODS18 particles were made into a uniform coating by melting. The static WCA of this coating was about 166° and water roll-off angle was <6°. This observation clearly points out that, mechanical damage of the SH powder affects roughness only and that the chemical network is intact.

Chemically, octadecyl chain grafted silica particles consist of urethane/urea links through which these chains orient themselves around the silica particles. Alkyl chains are wired together through van der wall’s interactions aided by the urethane hydrogen bonding. The strongly hydrogen bonded alkyl chains (*via* urethane linkages) provide low surface energy to the material which is essential to display superhydrophobic property. The flexible octadecyl chains move around the urethane bonds on melting by breaking the wander walls forces but not necessarily the H-bonds. The urethane linkages and physical interactions resist to a great extent these damages and help retain the chemical structure intact. However, on mechanical treatment, the labile aliphatic chains move away from surface, exposing the polar urethane moieties to water droplets. This is the rationale for the immediate reduction of SH features just after the water impalement test/hot water test. On heating or maintaining the damaged surface at ambient conditions, the chains become labile and will rise to the surface (as the urethane groups are liberated from the clutches of H-bonding with external water molecules). The mechanism of regenerative SH is schematically shown in Fig. 5. The arrangements of crystalline and projected aliphatic chains on fresh surface (assembly), damaged surface (more disturbed, facile access to polar urethane groups) and regenerated surface (partially re-assembled, but restricted exposure of polar groups) are illustrated. In addition, the SH coating repels the common stains like tea, coffee, milk and ink effectively. These stains on the coating got rolled-off with sliding angle <10° and contact angle > 150° (*S11*). The coatings was found to be superhydrophobic to high viscous liquids like glycerol and carboxymethyl cellulose salt dissolved in water (*S12*). These studies implies the utility of material from the societal point of view also.

We illustrated the concept of functionalising silica nanoparticles using long alkyl chains to enable melt-processable SH surfaces. A macro-crack healable, chemically and mechanically durable bulk superhydrophobic material with self-cleaning and self-regenerating features was demonstrated. By varying the grafting density of surface units, SH materials capable of melting at various temperatures can be explored. Novel SH systems can be developed by varying the chain length of grafted alkyl groups over the surface. It will be interesting to investigate the impact of the size and shape of silica nano particles on the SH behaviour of the resultant graft systems. High reactivity of silanes (long alkyl chain containing silanes) towards silanol group is also a viable route for the preparation of highly grafted SH particles which may result in melting class SH materials. Also, large area SH coating can be explored by powder coating of the present system. The approach can be extended to different micro-nanomaterials (like metal oxides and others) also for achieving the aforementioned properties which was considered as ‘unachievable’ hitherto in a single material.

## Methods

### Procedure for grafting of octadecyl chains on silica surface and preparation of SH coating

The dried silica nanoparticles (2.0 g) in triethylamine (30 ml) were ultra-sonicated for half an hour at 60 °C under N_2_, in presence of dibutyltindilaurate (1.0 wt. %). The mixture was taken in a round bottom flask and the octadecyl isocyanate (ODI) (10.0 g) was introduced into the solution drop by drop under nitrogen flow. Then, the temperature was increased to 80 °C and the grafting was carried out for 18 h. The grafted silica nanoparticle was separated by filtration and purified by repeated washing with toluene, acetone and distilled water. The final pale yellow coloured particles were obtained after drying under vacuum at 70 °C for 24 h. For the preparation of SH coating, firstly, the particles were ground well and ultra-sonicated at 60 °C for 2 h in xylene solvent (10 wt. % SH powders). The colloidal solution was dropped over a glass substrate and slowly heated and maintained at 150 °C for 8h. During this process, the particles melted and formed a coating.

## Additional Information

**How to cite this article**: Ramakrishna, S. *et al.* A robust, melting class bulk superhydrophobic material with heat-healing and self-cleaning properties. *Sci. Rep.* **5**, 18510; doi: 10.1038/srep18510 (2015).

## Supplementary Material

Supplementary Information

Supplementary Video 1

## Figures and Tables

**Figure 1:**
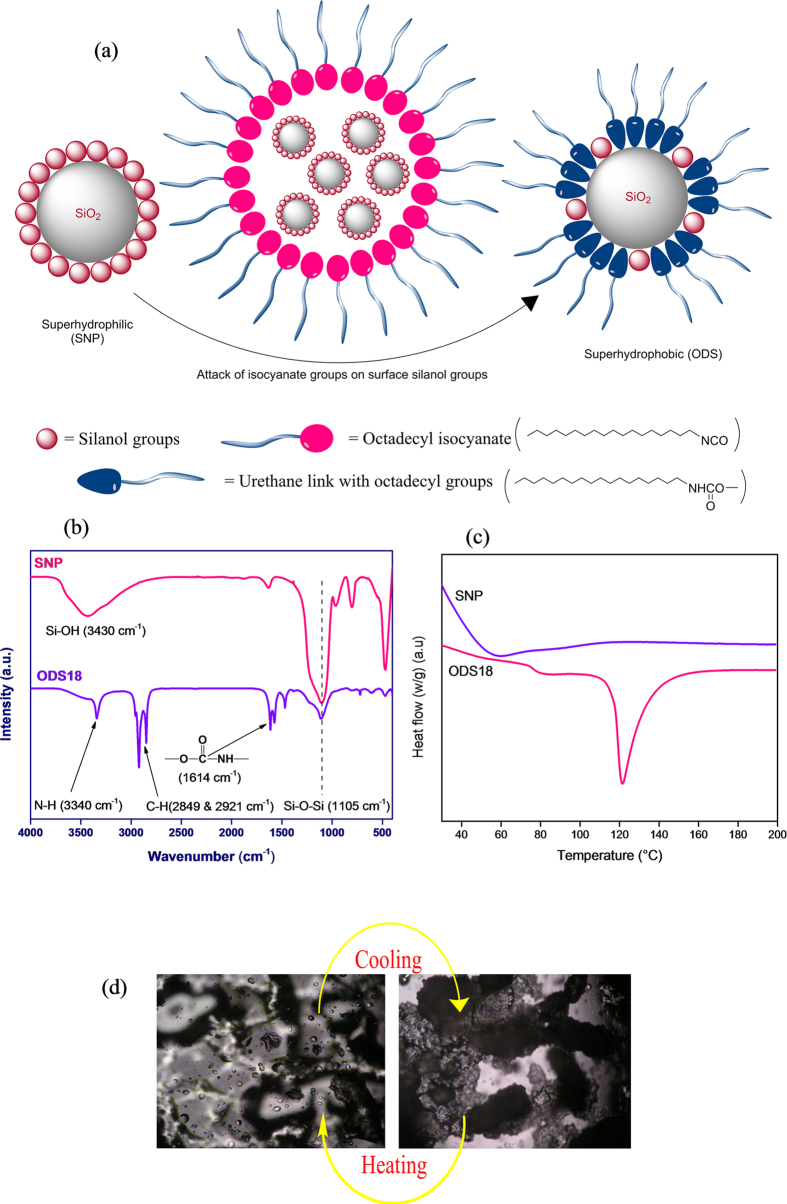
(**a**) Schematic representation of preparation of superhydrophobic silica particles by grafting of octadecyl chains on silica particles (**b**) FT-IR spectra of unfunctionalized silica nanoparticles (SNP) and ODS18 (**c**) Differential scanning calorimetry profiles of SNP and ODS18; ODS18 shows melting endotherm at 121 °C with a melting enthalpy of 137 J/g (**d**) Optical microscopic images of ODS18 particles on heating and cooling.

**Figure 2:**
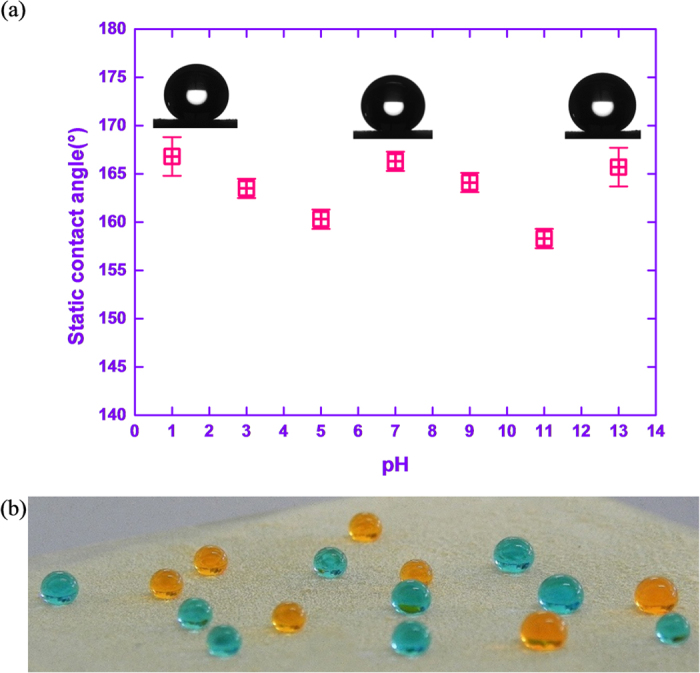
(**a**) Static WCA on ODS18 coating at different pH (**b**) The acidic (pH = 1; blue) and basic (pH = 13; orange) water droplets on ODS18 coating (coloured for visualization).

**Figure 3:**
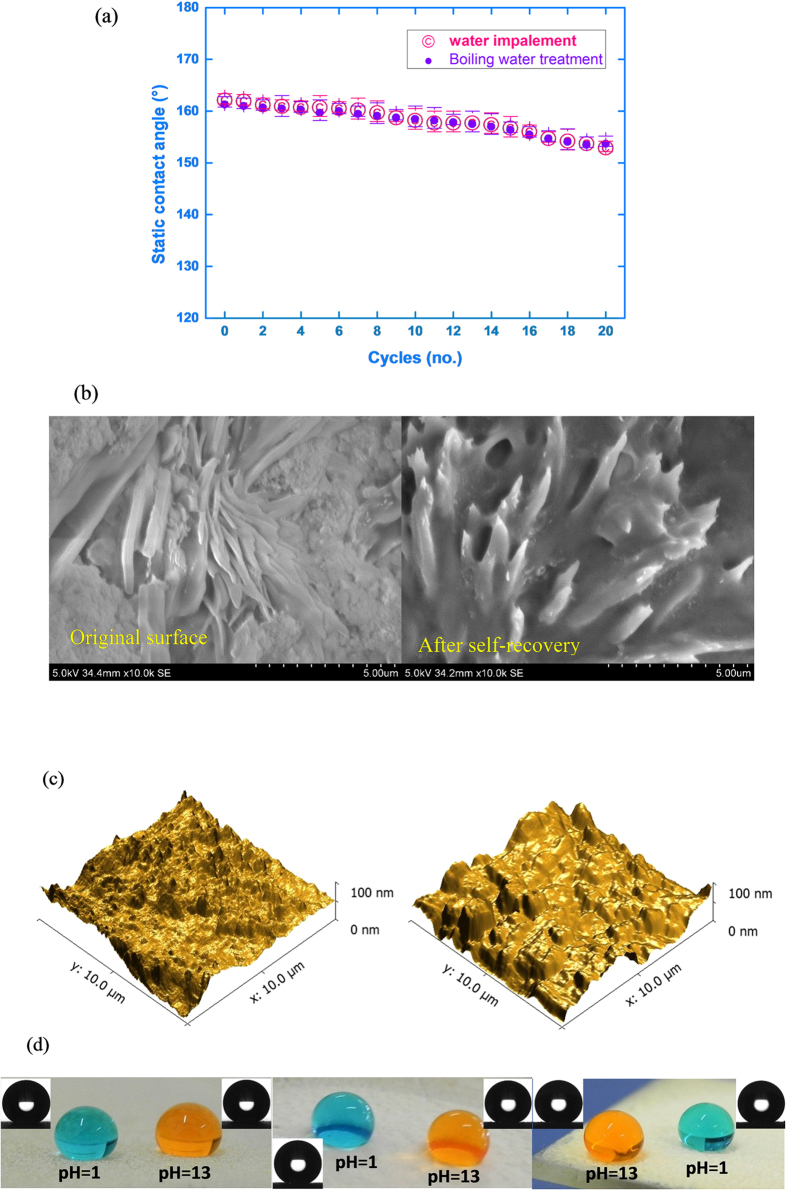
(**a**) Static WCA of coating after self-recovery from cyclic water impalement and cyclic boiling water treatments (**b**) The FESEM morphologies of coating before impalement (left) and after self-recovery (right) (**c**) The AFM images of coating before water impalement (left) and after self-recovery (right) (**d**) Optical images of water droplets over ODS18 surface before water impalement (left), self-recovered surface after 1h at ambient conditions (middle) and self-recovered surface after 5 minute heat treatment (right).

**Figure 4:**
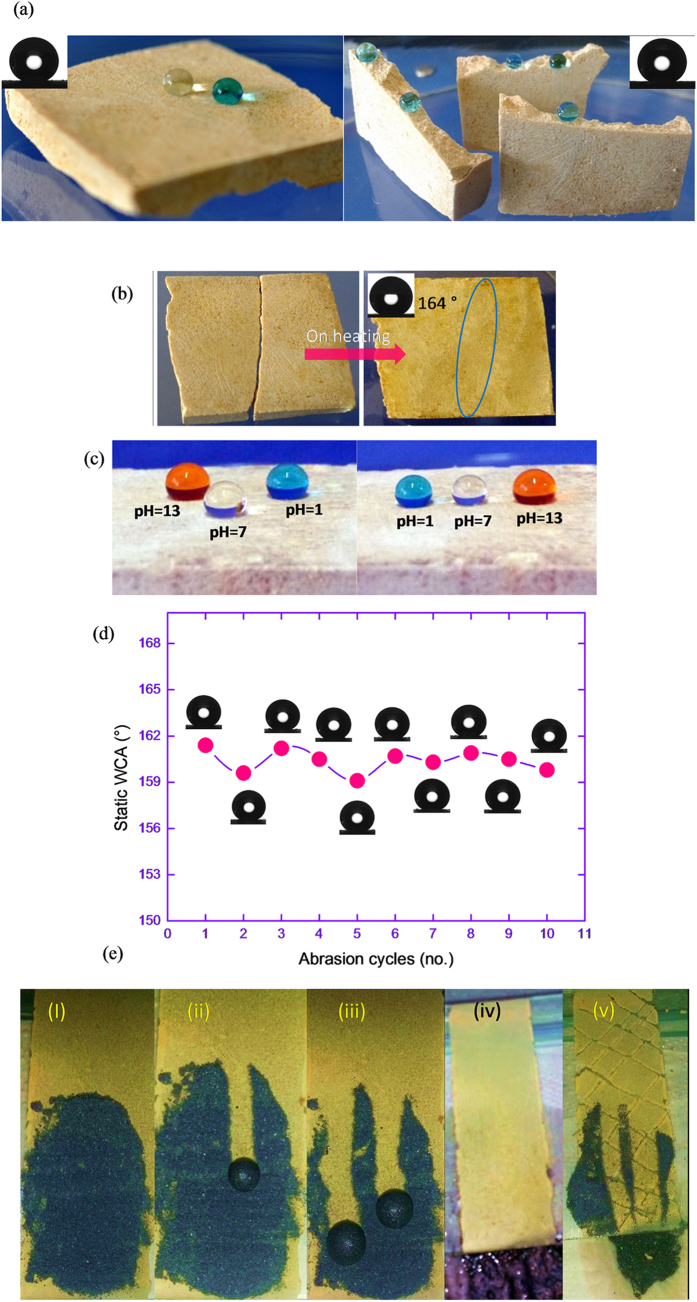
(**a**) Optical images of water droplets on ODS18 material: on the surface (left) and on the broken pieces (right) (inset: static WCA on the bulk surface (left); static WCA on the broken pieces (right) (**b**) Broken pieces (left); heat-healed surface (right) (**c**) Profile of water droplets (pH = 1, pH = 7 and pH = 13) on original surface (left), and on healed area (right) (**d**) Static water contact angle of abraded surfaces of bulk material (**e**) (i–iv); demonstration of self-cleaning sequence on the ODS18 surface using magnesium powder and (v) self-cleaning on cut SH surface.

**Figure 5:**
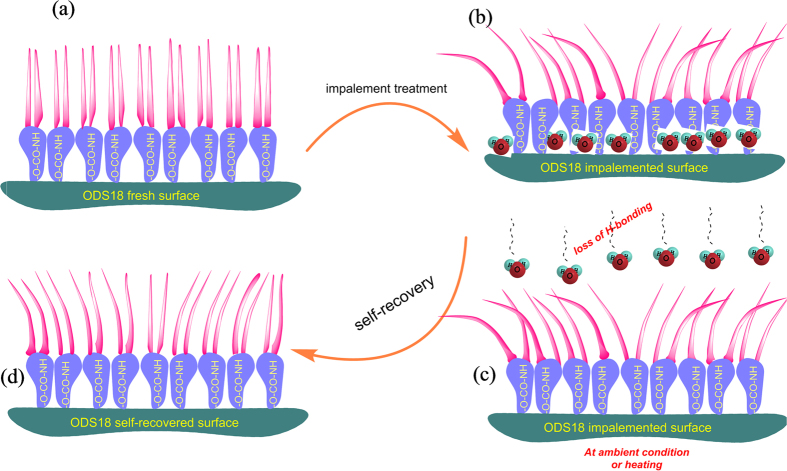
Schematic representation of proposed self-regeneration mechanism of ODS18 surface (**a**) self-assembled alkyl chains on original coating (**b**) distorted chains on impalement/similar tests (**c**) release of hydrogen bonded water molecules and subsequent evaporation (**d**) re-self-assembled alkyl chains at ambient temperature or on heating.
