# Supplementary material for: A robust, melting class bulk superhydrophobic material with heat-healing and self-cleaning properties
Source: Sci Rep. 2015 Dec 18;5:18510. doi: 10.1038/srep18510 (PMC4683526; doi:10.1038/srep18510)
Supplement: Supplementary Information [file srep18510-s1.pdf]

A robust, melting class bulk superhydrophobic material with heat-healing and self-cleaning properties

Ramakrishna S., Santhosh Kumar K.S.*, Dona Mathew, Reghunadhan Nair C.P.

Polymers and Special Chemicals Group, Vikram Sarabhai Space Centre, Thiruvananthapuram-22,

E-mail: santhoshkshankar@yahoo.com

Supporting Information

S1

Materials: Silica nanoparticles (20-40 nm diameter) Sigma-Aldrich, Germany) was used after drying at 125 °C for 12h under vacuum. Octadecyl isocyanate (98%, Sigma-Aldrich, USA), triethylamine (99%, Fisher Scientific, India), dibutyltindilaurate (95%, Sigma-Aldrich, Germany), toluene (99%, Central Drug House, India) and acetone (99%, Sisco Research Laboratory, India) were used as received without further purification.

Instrumental techniques: Perkin Elmer Spectrum GX-A FTIR spectrometer, Finnigan EA 1112 Series Flash Elemental Analyzer TA instruments model 2960 (TGA), Systronics 362 pH meter Hitachi SU6600 variable Pressure Field Emission Scanning Electron Microscope (FESEM) were used for respective analyses. Specific surface area of samples (BET isotherm) was determined by Quantachrome NOVA 1200e surface area analyzer. Data Physics contact angle instrument OCA-15EC with SCA 20 software was used to determine the static contact angles (Young-Laplace method) of surfaces. Fluid drops of 5μl was placed at five different positions on the surface of coating and is reported here. The surface roughness of coatings was analyzed by Agilent Technologies 5500 Atomic Force Microscope unit. X-ray diffraction (XRD) patterns were recorded

on a Bruker D8 discover diffractometer. Nano particles were analysed by XRD Scan speed increment of 0.01° and time of step 0.5 second was selected for analysis. The adhesion test was conducted using ASTM grade M250 adhesive tape. A 1×1 cm^2 area of coating was selected for testing and this area was further subdivided into several square portions by making perpendicular lines over SH coating. Then, the cut area was adhered with the M250 grade adhesive tape and pulled-off in a single pull. The number of squares detached from the coating was counted and reported in percentage.

We attempted to detach the grafted moiety from silica surface for assessing grafting density by Barendson equation or TGA method, but could not succeed due to the insolubility of ODS18 in different solvents (even after ultrasonication) like dimethyl formamide, dimethyl acetamide, N-methyl pyrrolidone, hexane, tetrahydrofuran, toluene and acetone. The observed grafting yield in ODS18 was ~ 74 % from TGA. Here, we tried to obtain the grafting in a semi-empirical route.

Theoretically:

Radius = 0.162 nm (assumption: O-Si-O is symmetrical)

Surface area= $4\ \pi r^2 = 0.33\ nm^2$

Average area occupied by one Si-O group= $0.33\ nm^2/4= 0.08\ nm^2$

OH group density = $1/0.08= 12.5$ *groups/*nm^2

Here, the nanoparticle radius varies between 10-20 nm.

If r=10 nm= $\mathbf{10\ x10^{-7}}$ ***cm***

Surface area= $4\pi r^2\ = 4x3.14x10^{-12}\ cm^2 = 0.3x\ 10^{-11}\ cm^2\ \ = 12x\ 10^2\ nm^2$

Volume of a particle = $(4/3)\ \pi r^3 =\ (4/3)\ x\ 3.14\ x10^{-18}\ cm^3$

Weight of one particle = density x volume= $8.79\ x\ 10^{-18}$ *g =* $8.8\ x\ 10^{-18}$ *(density =2.1 g/cc)*

Extent of grafting here is 74 % i.e. 25 g particle contains 74 g graft.

Assume, molecular weight of single graft = 295g/mole (however, excess octadecyl isocyanate attacks urethane linkages and extends the chain at least to two alkyl units on one graft).

No. of moles of graft on 25 g of nanoparticle = 75/295=0.25 moles = $0.25x\ 6.023x\ 10^{23}$ *molecules*

No. of particle of silica = $25/\ (8x10^{-18}) = 3x\ 10^{18}$

No. of graft per particle = 0.25x 6.02x 10^{23}*/ (3x* 10^{18}*) = 6x*10^3 *= 48000*

Grafting areal density =48x10^3*/ (1200* nm^2*) =* **40 grafts per** $\mathbf{nm^2}$

The calculated grafting density is higher than the theoretical because of two reasons:

1. The calculation assumes all particles are spherical. All other shapes have more surface area than a sphere. The SEM images support that grafted particles are not in spherical shape.
2. It assumes also that each graft is composed of one single unit of alkyl groups, while there is possibility for already formed urethane to react with excess isocyanate to form allophanate groups. This is particularly true when the alcohols are least reactive as in the present case. This also can increase the extent of grating and graft molecular weight. (*Silanols are somewhat less reactive than even carboxylic acid as its nucleophilicity is questionable in view of the nπ-dπ back bonding that reduces the nucleophilicity of the oxygen atom. In such a case, reaction of already formed urethanes with an incoming isocyanate is equally feasible).*

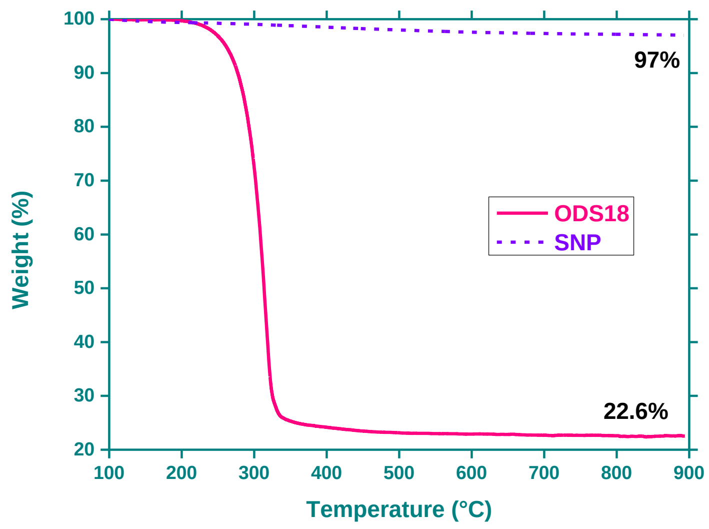

Thermogravimetric analysis of bare silica nanoparticles (SNP) and alkyl chains grafted silica particles (ODS18)

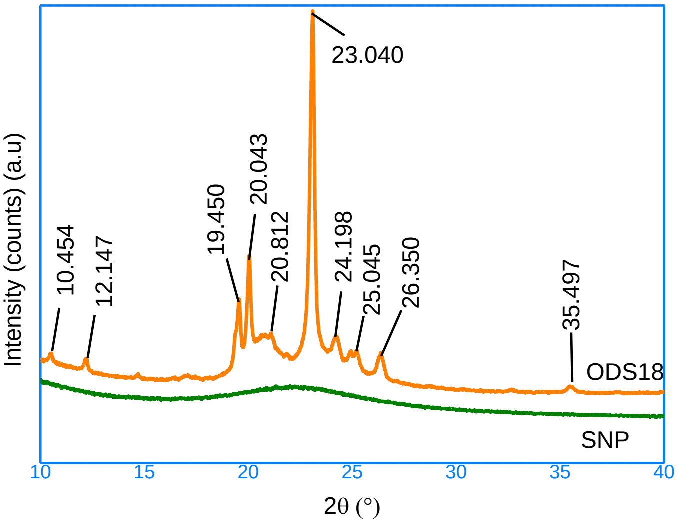

S2 XRD patterns of ODS18 particles

S3 Video of rolling/bouncing water droplets on SH surface from contact angle unit.

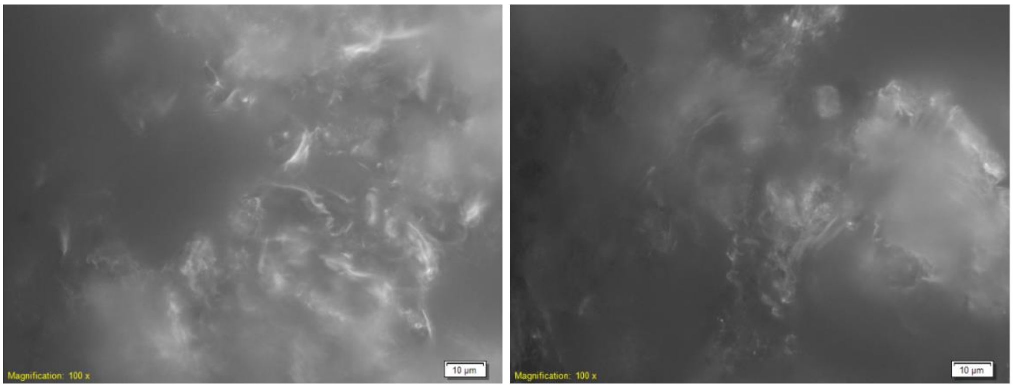

S4 Optical images of ODS18 coating (a) before impalement (b) after self-recovery

(shining crystals are retained)

S5 Calculation of water hammer pressure:

The water hammer pressure created by the water droplets is given by (*Ref: Y. C. Huang, F. G. Hammitt and W. J. Yang, Journal of Fluids Engineering, 1973, 95, 276-292).*

The velocity of water droplet V = $\mathbf{(2gh)}^{1/2}$ (for freely falling body)

$$\textit{(Initial (potential energy+ kinetic energy) =Final (PE +KE)}$$

$$mgh + 0 = 0 + (0.5\, mv^{2})$$

$$\text{i.e. } mgh = 0.5\, mv^{2}, \text{ hence } V = (2gh)^{½}$$

$$\underline{\textbf{Here } V = 3.13 \textbf{ m/s} \text{ (i.e. } g = 9.8 \text{ m/s}^{2}, h = 0.5 \text{ m)}}$$

Water hammer pressure, $P_h = 0.2\, \rho\, C\, V$

Where ρ= density of water = 1000 kg/m^3

C= velocity of sound in water at 28 °C =1500 m/s

V= velocity of water = 3.13 m/**s**

Water hammer pressure, P = 0.2(1000) (1500) (3.13) =939 kPa = ~ 1 MPa

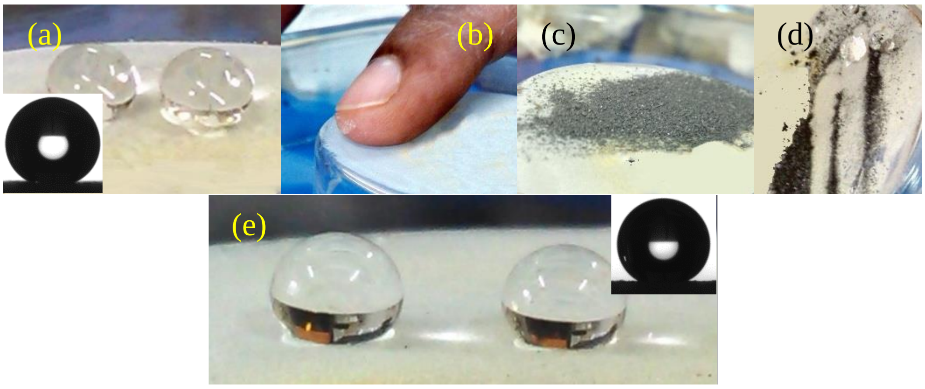

S6 Optical images of (a) water droplets on ODS18 surface (162°) (b) finger rubbing on ODS18 surface (c) polluted with magnesium powder on ODS18 surface after finger rubbing and then heated at 80 °C, 5min (d) self-cleaned surface after heat treatment and (e) self-recovered ODS18 surface (160°) (inset shows optical images from contact angle unit).

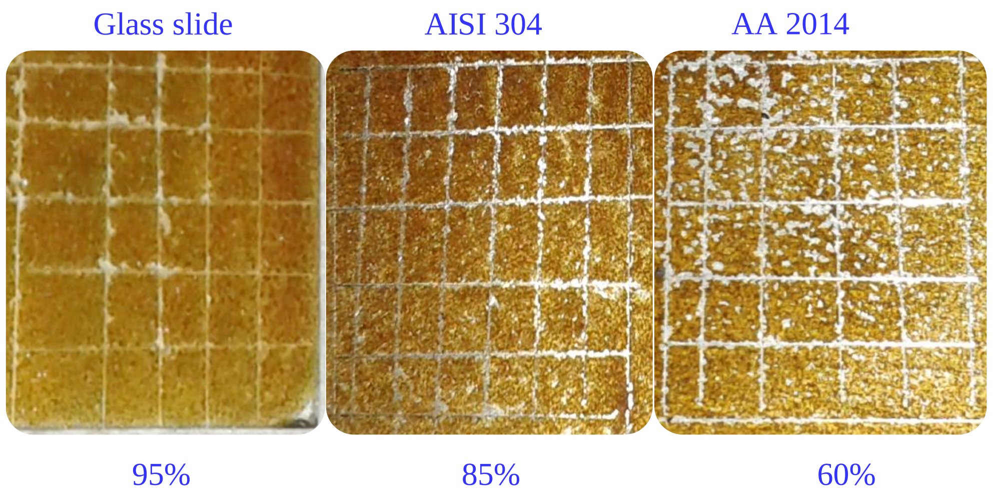

S7 Photographs of ODS18 coating after cross-hatch test on glass (left), SS (middle), aluminium (right)

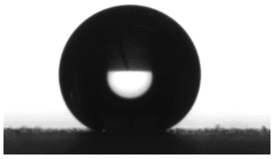

S8 Optical image of water droplet on ODS18 coating after one year exposure (storage) at ambient condition (static WCA 163±2°)

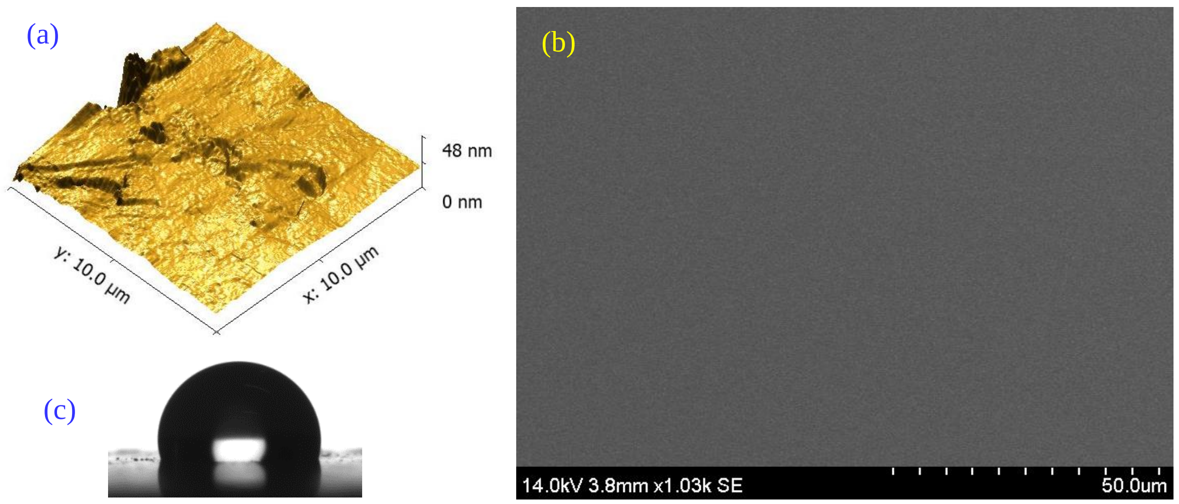

(d)

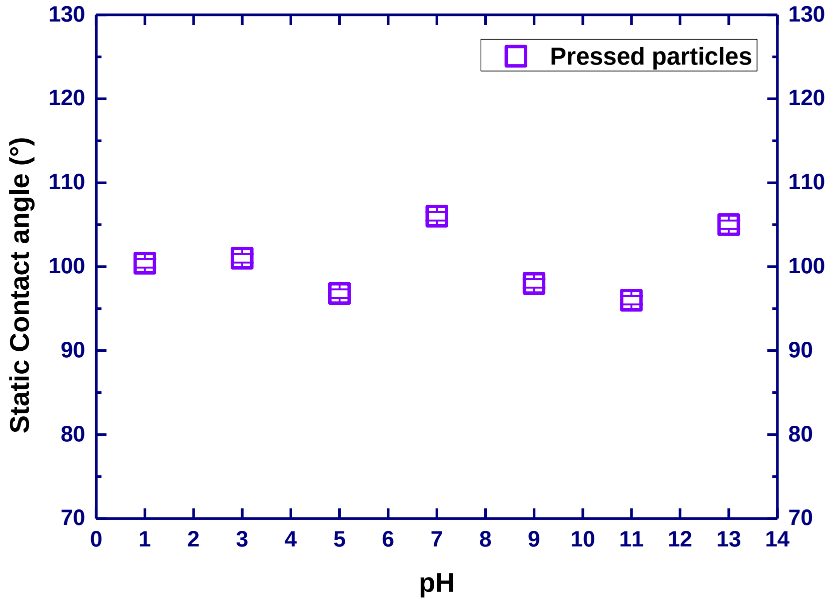

S9 (a) AFM image shows very low surface roughness on pressed surface (<10nm) (b) FESEM image shows absence of any distinct morphology on the pressed surface (c) Optical image of water droplet on pressed surface (106°) (d) variation in WCA on pressed surface as a function of pH.

S10 Air fraction of the coating (ODS18) was calculated by using the equation as follows;

a) $\text{Cos}\ \theta_c = f_1 \text{Cos}\ \theta - f_2$

b) $f_1 + f_2 = 1$

Where θ_c = Contact angle on SH surface; f_1 = fraction of solid surface wetted by liquid; θ = Contact angle on similar smooth surface and f_2 = air fraction surrounded by the liquid droplet

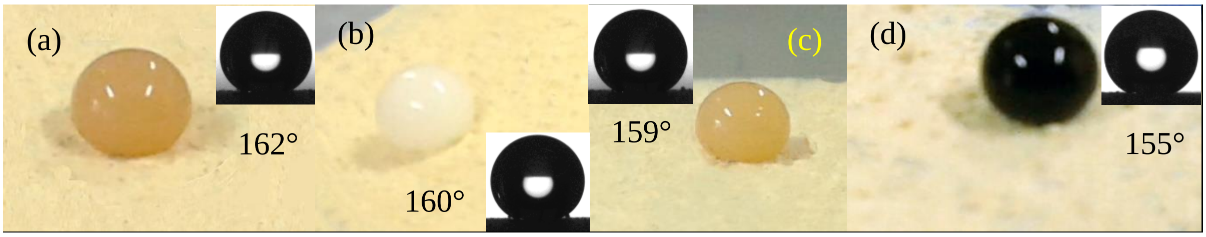

S11 Optical images of (a) tea (162°), (b) milk (160°), (c) coffee (159°) and (d) ink (155°) on ODS18 surface (inset- optical images from contact angle unit)

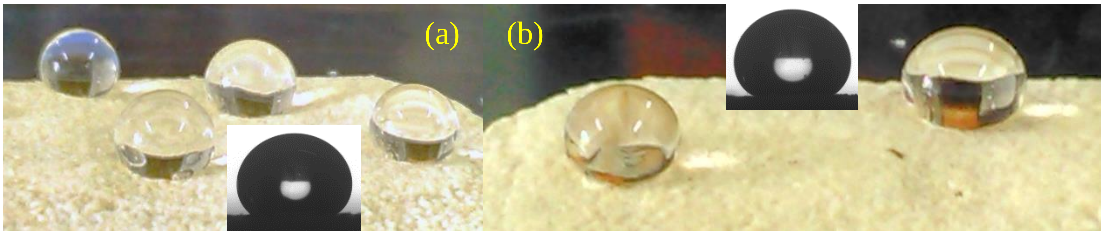

S12 Optical images of high viscous liquids (a) aq. carboxymethylcellulose sodium salt solution (CA= 153°) and (b) glycerol (CA= 155°) on ODS18 surface (inset shows optical images from contact angle unit).
